# Evaluation of Claw Lesions in Beef Cattle Slaughtered in Northern Portugal: A Preliminary Study

**DOI:** 10.3390/ani14030514

**Published:** 2024-02-04

**Authors:** Mafalda Seixas, Dina Moura, Luca Grispoldi, Beniamino Cenci-Goga, Sónia Saraiva, Filipe Silva, Isabel Pires, Cristina Saraiva, Juan García-Díez

**Affiliations:** 1 Department of Veterinary Sciences, School of Agricultural and Veterinary Sciences, Universidade de Trás-os-Montes e Alto Douro, Quinta de Prados, 5000-801 Vila Real, Portugal; mafaldaseixas.10@hotmail.com (M.S.); fsilva@utad.pt (F.S.); ipires@utad.pt (I.P.); crisarai@utad.pt (C.S.); 2 Divisão de Intervenção de Alimentação e Veterinária de Vila Real e Douro Sul, Direção de Serviços de Alimentação e Veterinária da Região Norte, Direção Geral de Alimentação e Veterinária, Lugar de Codessais, 5000-421 Vila Real, Portugal; dina.moura@dgav.pt; 3 Dipartamento di Medicina Veterinaria, Universitá degli studi di Perugia, 06126 Perugia, Italy; grisluca@outlook.it (L.G.); beniamino.cencigoga@unipg.it (B.C.-G.); 4 Faculty of Veterinary Science, Department of Paraclinical Sciences, University of Pretoria, Onderstepoort 0110, South Africa; 5 Veterinary and Animal Research Centre (CECAV), University of Trás-os-Montes e Alto Douro, Quinta de Prados, 5000-801 Vila Real, Portugal; soniasarai@gmail.com; 6 Associate Laboratory for Animal and Veterinary Science (AL4AnimalS), 1300-477 Lisboa, Portugal

**Keywords:** beef cattle, claw lesions, welfare, carcass, slaughterhouse

## Abstract

**Simple Summary:**

While most research on claw diseases focuses on dairy cattle, this study aimed to evaluate the prevalence of claw disorders in beef cattle in northeast Portugal. The investigation was an observational study carried out at two slaughterhouses, in which claw lesions were assessed according to the ICAR Claw Health Atlas. The influence of sex and age and the potential economic impact on hot carcass weight, carcass classification, and fat coverage were investigated. The results revealed a high animal prevalence of claw lesions (65.8%), with the primary lesions being of a non-infectious mechanical nature, including heel horn erosion, double sole, and asymmetric claws. The lesions found are consistent with the production method in the area under study, where beef cattle are raised in small, rustic premises with uneven floors and beds made of a mix of manure and plant material. Also, the impact of claw lesions on carcass characteristics (weight, classification, and fat deposition) was not evident. Thus, the presence of claw lesions in beef cattle raised under the conditions of this geographical area does not seem to cause a negative impact on both animal health and the farm economy.

**Abstract:**

Claw diseases have a profound impact on cattle welfare, affecting behaviors such as grazing, rumination, rest, decubitus, and water consumption. This study aimed to assess the prevalence of claw lesions and classify them according to the ICAR Claw Health Atlas (International Committee of Animal Recording) in two slaughterhouses. The influence of claw lesions on carcass weight, classification, and fat deposition was also examined. Involving 343 crossbreed cattle from 103 different extensive or semi-intensive farms, this study found an animal prevalence of claw disorders at 65.8%, with a higher incidence in females (n = 207, 60.35%) compared to males (n = 136, 39.65%). Despite the observed prevalence, claw lesions were not influenced by age or sex (*p* > 0.05). The main claw lesions identified, including heel horn erosion, double sole, and asymmetric claw, were consistent with the cattle management practices in the study area. These cattle were raised in small, rustic premises with uneven floors, utilizing a mix of manure and plant material as bedding and lacking access to pasture. Also, no negative economic impact was detected concerning carcass weight, classification, or fat deposition. Consequently, it was concluded that the presence of claw lesions in beef cattle raised under the characteristic management of this geographical area does not adversely affect animal health or farm economics.

## 1. Introduction

Claw lesions in cattle are a group of diseases responsible for causing pain and locomotor disturbance, leading to a loss of well-being [1]. Therefore, claw pathologies result in significant economic losses in both dairy and beef cattle [2], either due to a reduction in milk production, decreased carcass yield, or the cost of antibiotic treatments [1]. The decrease in body condition is associated with a reduced voluntary food intake, owing to greater difficulty in moving to feeders or grazing areas. Furthermore, in cases of severe lameness, culling might be necessary, leading to subsequent economic loss. The risk factors for the occurrence of claw lesions are associated with the intrinsic characteristics of the animal as well as external factors [3]. Furthermore, a survey related to bull beef quality in the United States concluded that a high percentage of carcasses from cattle affected by claw lesions had inadequate carcass conformation, arthritic joints, and carcass bruises, decreasing their commercial value [4].

Within the realm of bovine characteristics, factors including body weight, claw conformation, hoof dimensions, hormonal fluctuations, metabolic variations, claw color [3,5,6,7], and the presence of a cow-hock posture have all been proposed as potential risk contributors to claw lesions. These factors influence the distribution and alteration of forces within the hoof structure [8]. Hormonal and metabolic shifts, notably those occurring during calving, are theorized to influence changes in the connective tissues supporting the pedal bone (suspensory apparatus) within the hoof wall, ultimately leading to instability in the distal phalanx [9]. Furthermore, the calving process can lead to a thinning of the digital cushion, compounded by alterations in the suspensory apparatus, as previously elucidated. Malformed claw conformation can trigger a redistribution of weight, potentially amplifying biomechanical forces during limb loading [10].

Ensuring proper weight distribution across the claws is a central goal of routine foot trimming aimed at preempting claw ailments [11]. Furthermore, suboptimal postures, such as the ‘cow-hock’ stance, can exert profound influences on the biomechanical forces impacting the hoof structure. Regarding males, claw lesions lead to a decrease in ejaculated semen volume and quality, along with diminished spermatozoa viability and longevity. Moreover, it impairs the leaping prowess, particularly in instances where the most injurious lesions manifest in the posterior extremities, thereby attenuating the productive tenure of the bull [12].

Addressing external influences, variables encompassing nutritional input [5], season [13], bedding hygiene practices [14,15], and the lack of implementation of claw-trimming prophylactic schemes [16] have been delineated as contributing risk factors in the pathogenesis of lameness.

In dairy farms, the surveillance of locomotion and hoof conditions occurs with greater frequency, thus facilitating the initiation of interventions at an earlier stage. In the context of beef cattle production, factors such as shorter cattle lifespans, fewer human–animal interactions leading to reduced hoof issue monitoring, and the inherent difficulties in executing trimming schemes due to temperamental traits collectively amplify the susceptibility to claw lesions.

Cattle production in the northeast of Portugal is characterized by small-scale farming, where calves are raised in small, rustic stables with uneven floors. The bedding consists of a mixture of manure and plant material, which is usually removed twice a year. Calves are kept indoors in freestalls but do not have access to pasture areas. Additionally, restraint equipment in this geographical area is generally scarce, making the periodic assessment of hoof health nearly nonexistent [17].

Given the lack of available information on hoof pathology in the studied area and considering its association with significant economic losses, the objective of this study was to characterize the types of hoof lesions in animals intended for human consumption, determine the prevalence of these lesions, and assess whether their presence has a negative economic impact by influencing weight, classification, and fat deposition in the carcass.

## 2. Materials and Methods

### 2.1. Cattle and Claw Disorders Characterization

The present work evaluated the main claw lesions in beef cattle slaughtered in two slaughterhouses in the northeast of Portugal. A total of three hundred and forty-three carcasses (343) were examined over a period of six months (September 2022 to March 2023). Data regarding breed, sex, age, and carcass data (hot carcass weight, fat coverage, and carcass conformation) were recorded. 

The claw lesions were classified according to the Claw Health Atlas published by the International Committee of Animal Recording (ICAR) [18] as asymmetrical claws (AC), concave dorsal wall (CD), corkscrew claw (CC), digital dermatitis (DD), interdigital/superficial dermatitis (ID), double sole (DS), heel horn erosion (HHE), axial horn fissure (HFA), horizontal horn fissure (HFH), vertical horn fissure (HFV), interdigital hyperplasia (IH), interdigital phlegmon (IP), scissor claws (SC), sole hemorrhage diffused (SHD), sole hemorrhage circumscribed (SHC), swelling of coronet and/or bulb (SW), sole ulcer (SU), bulb ulcer (BU), toe ulcer (TU), toe necrosis (TN), thin sole (TS), white line fissure (WLF), and white line abscess (WLA). Claw lesions assessment (based on ICAR guidelines) was carried out at the slaughterhouses by two veterinarians with over 20 years of experience in cattle medicine. For statistical purposes, affected cattle are considered those that presented a claw lesion, regardless of the number of claws affected. 

Information on carcass classification, obtained from slaughterhouse records, was carried out according to the SEUROP grip, in which conformation is assessed on an S to P basis as defined in European law [19]. Regarding the fat cover, the cattle sampled were classified into three categories based on the 1 to 5 grid defined by law [19] as follows: low-fat coverage (including classes 1-low and 2-slight), medium-fat coverage (including class 3-average), and high-hat coverage (including classes 4-high and 5-very high).

### 2.2. Data Analysis 

All data were entered into an Excel database, and statistics were carried out using Jamovi^®^ [20,21]. The prevalence of claw lesions is presented as animal prevalence (total affected cattle sampled by at least one claw lesion/total cattle sampled). Also, we investigated by Chi-squared test (χ^2^) if age, sex, breed, hot carcass weight, fat coverage, and carcass classification are influenced by the presence or absence of claw lesions. Statistical significance was set at *p* < 0.05.

To assess the influence of the presence or absence of claw lesions on hot carcass weight, carcass classification, and fat coverage, sampled cattle were categorized by age and weight in several categories, as follows: weight ≤ 100 kg, >100 ≤ 150 kg, >150 ≤ 200 kg, >200 ≤ 250 kg, >250 ≤ 300, >300 ≤ 350 kg, >350 ≤ 400 kg; age ≤ 6 months, <7 ≤ 12 months, <13 ≤ 18 months, >18 months.

## 3. Results

### 3.1. General Results

The sampled cattle (n = 343) originated from 103 different extensive or semi-extensive farms intended for beef production. Sampled cattle were composed exclusively of crossbreeds, comprising 207 females and 136 males, with an average age of 10.5 ± 3.43 months (ranging from 5 to 32 months). Carcass classification, as per the SEUROP grid, exhibited the following distribution: E (1; 0.3%), U (7; 2.3%), R (63; 20.8%), O (229; 75.6%), P (3; 0.99%), and E (0; 0%). With regards to fat coverage assessment, results showed that 1 (0.3%), 46 (13.4%), 294 (85.7%), and 2 (0.6%) carcasses were classified as 1, 2, 3, or 4, respectively. Notably, no carcass achieved a classification of 5 concerning fat coverage.

### 3.2. Cattle Claw Disorders 

The prevalence of claw lesions (i.e., animal prevalence) (Table 1) in the sample studied was 65.8%, being higher in females (130, 37.90%) than in males (96, 27.98%). Among cattle with claw lesions (n = 226), 94.2% of them displayed lesions in the forelimbs, while only 14.5% presented lesions in the hindlimbs. Most cattle presented lesions in either one (19.4%) or two limbs (74.0%), predominantly characterized by a singular type of lesion. (79.3%). A total of 204 (89.87%), 181 (79.74%), 26 (11.45%), and 22 (9.69%) displayed lesions in the left forelimb, right forelimb, left hindlimb, and right hindlimb, respectively. A total of 43 and 171 cattle presented lesions in one or two forelimbs, while 18 and 16 presented lesions in one or two hindlimbs, respectively. Thus, 214 (94.27%) and 34 (14.97%) cattle displayed claw lesions in the fore or hindlimbs, respectively. In addition, 44 (19.38%), 169 (74.75%), 5 (2.20%), and 9 (3.96%) of the sampled cattle presented lesions in one, two, three, or four limbs, respectively.

The prevalence and location of claw lesions according to the ICAR classification are presented in Table 2. Among the 21 lesions listed in the ICAR atlas, only 11 of them were observed, with double sole and heel horn erosion, being the most frequent claw lesions. Additionally, claw lesions were mainly located in the forelimbs (*p* < 0.05).

Lesions regarding corkscrew claws (CC), interdigital hyperplasia (IH), scissor claws (SC), sole hemorrhage (SH), swelling of coronet and/or bulb (SW), thin sole (TS), and white line diseases (WL) (including fissures (WLF) and abscesses (WLA)) were not observed. A descriptive analysis of the distribution and prevalence of each claw lesion by sex, age, hot carcass weight, carcass classification, and fat deposit was presented as Supplementary Material.

### 3.3. Influence of Sex and Age in the Presence of Claw Lesions in Cattle

**Figure 1:**
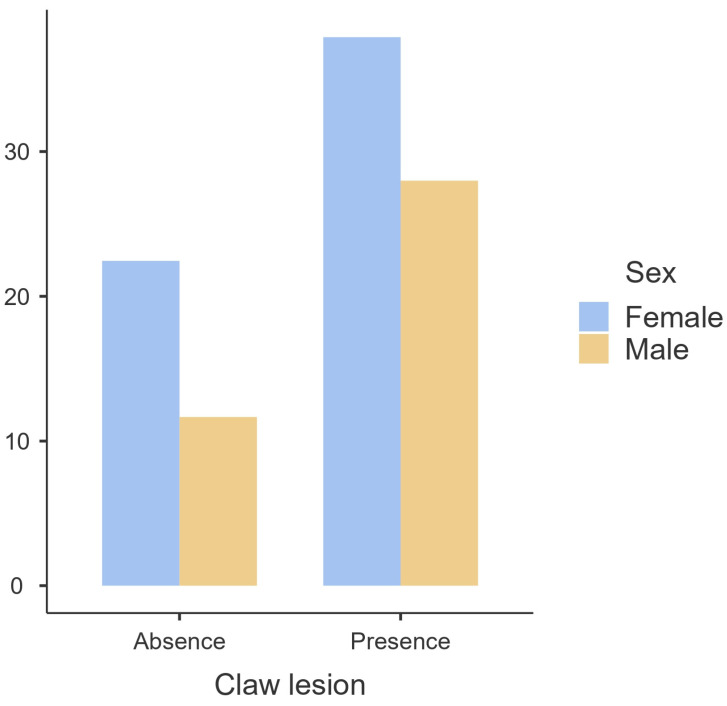
Prevalence (%) of claw lesions by sex.

**Figure 2:**
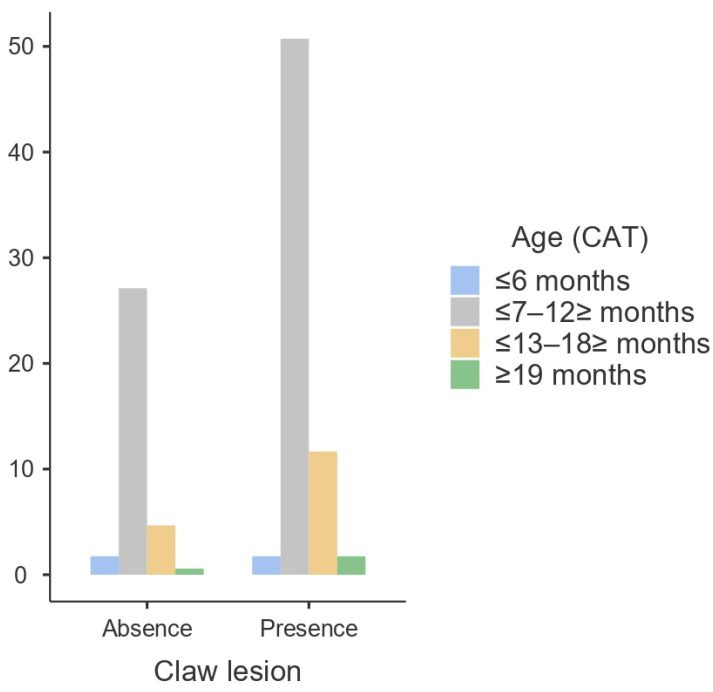
Prevalence (%) of claw lesions by age category (CAT).

**Figure 3:**
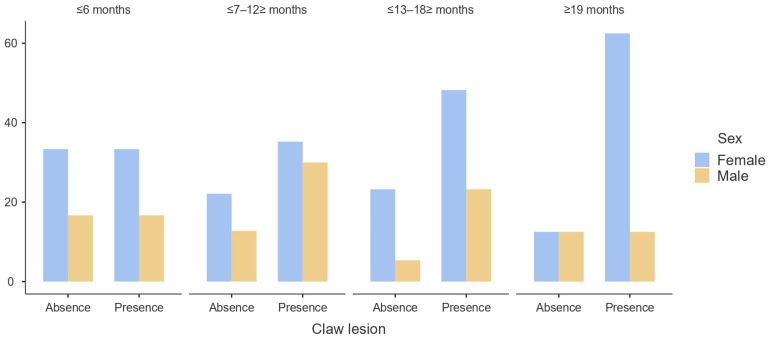
Prevalence (%) of claw lesions by sex and age category.

According to the results obtained, the presence of claw lesions does not appear to be influenced by sex (*p* > 0.05) (Figure 1) or age (*p* > 0.05) (Figure 2 and Figure 3). Regarding the carcass characteristics, the presence of claw lesions neither influences its classification nor the fat coverage (*p* > 0.05). The analysis of data by type of claw lesion showed that double sole and heel horn erosion have a higher prevalence in cattle between 6 and 18 months of age. Regarding hot carcass weight, given the heterogeneity of the sample, the influence of the presence of claw lesions on the final hot carcass weight, carcass classification, and fat coverage were studied by age and sex. A descriptive analysis of the influence of claw lesions on factors previously described is presented as Supplementary Material.

Sex had no influence on the presence of claw disorders (*p* > 0.05), although the results show a higher prevalence in females (Figure 1). Furthermore, there were no significant differences by age for sex (*p* > 0.05), but in females, the older the age, the higher the prevalence of claw lesions. In contrast, males displayed a lower prevalence of claw lesions as age increased (Figure 2). Regarding age, it also had no influence on the development of claw lesions (*p* > 0.05), although there is a sharp increase in prevalence from 7 months to 12 months (Figure 3). Although the group of cattle between 13 and 18 months has a higher prevalence, it decreases drastically compared to the previous category.

The carcass weight was not influenced by the presence of claw lesions (*p* > 0.05) (Table 3). Due to the heterogeneity of the sample, categorization by age and sex shows that cattle aged between 7–12 and 13–18 months with claw lesions have a higher carcass weight. 

**Figure 4:**
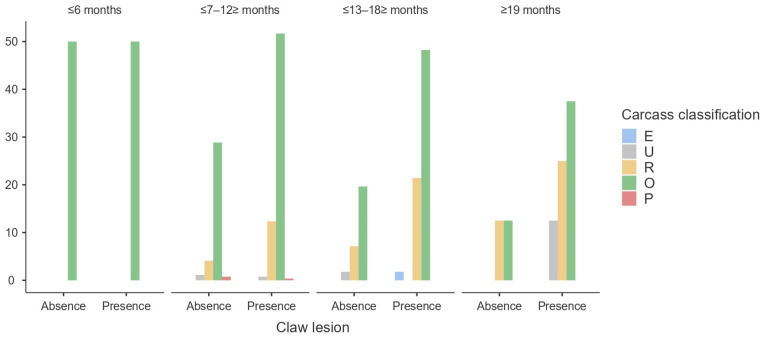
Distribution (%) of carcass classification by presence/absence of claw lesions.

Regarding the classification of carcasses, 96.79% were classified as R or O. Also, carcass classification was not influenced (*p* > 0.05) (Figure 4) by the presence of claw lesions.

## 4. Discussion

The presence of claw lesions in bovine livestock raises significant concerns for both animal welfare and economic sustainability. In our study, we observed an animal prevalence rate of 65.8% for podal lesions. Previous research on bovine claw lesions has primarily focused on dairy cattle due to the ease of hoof assessment. In dairy cattle, animal prevalence rates exhibit substantial variability, ranging from 25% to 80% [14,22,23,24,25,26]. Similarly, among beef cattle, the animal prevalence of claw lesions shows considerable disparity, ranging from 1% to 90% [16,27,28,29,30,31,32,33].

It is worth noting, however, that some researchers [34] have suggested that prevalence studies conducted in slaughterhouses may overestimate results, as not all lesions evoke clinical symptoms in the affected animals [24]. Therefore, in a study [35] where it was observed that 90% of the sampled cows did not show abnormalities in their gait, subsequent claw trimming revealed that 15.7% and 13.6% presented sole ulcers and heel horn erosion lesions, respectively. In accordance with lesion localization analysis, it was observed that nearly 95% of such lesions were predominantly located in the forelimbs, contrary to what was reported by other researchers [36], where a higher prevalence was documented in the hindlimbs. The forelimbs exhibit anatomical attributes that render them better suited for weight-bearing functions relative to the hindlimbs, which are primarily adapted for propulsion owing to their proximity to the body’s center of gravity [37]. Although elucidating the precise causative factors behind the observed results presents a challenge, plausible associations may be drawn with respect to the beef management practices within the study region as described elsewhere [17]. Briefly, the prevalent practice among the local agricultural community involves rearing calves under conditions of freestall in small-scaled premises with irregular flooring, in which mobility is restricted, thereby fostering an increased susceptibility to podal issues, primarily attributed to the substantial weight-bearing role of the front limbs. Since most premises have irregular flooring, lying behavior may be altered. Therefore, inappropriate housing conditions (reduced space and lack of bedding comfort) may lead to cows spending more time standing, which could explain the higher prevalence in the front limbs [38].

Furthermore, the preponderance of non-infectious lesions rather than purely infectious etiologies (e.g., digital/interdigital dermatitis) underlying the principal lesions identified may offer a cogent rationale for these findings. This fact has been referred to by some authors [24], who found that cattle housed displayed a high prevalence of claw lesions compared to cattle reared in pastures [14].

Regarding the types of lesions observed in our study, double sole, asymmetric claws, and heel horn erosion accounted for 94.3% of the total claw lesions. According to the published literature, the type of observed lesions, as well as their prevalence, varies widely due to differences such as herd size, production type, management, characteristics of the animals under study, and criteria for diagnosing claw lesions [31,39]. Therefore, establishing comparisons is challenging since our work is limited to observational surveillance of claw lesions, and these kinds of data, as previously indicated, cannot be assessed at the slaughterhouse. However, the fact that most of the lesions are of a mechanical nature rather than infectious is in accordance with the research available on claw lesions.

Regarding asymmetric claws, it has been observed that there are anatomical differences between the lateral and medial digits in calves [40], which means that the irregular distribution of the animal’s own weight may contribute to the development of this pathology. Some authors [25,41] have observed a prevalence twice as high as in our study for asymmetric claws, while other studies reported prevalences for concave dorsal walls 6-fold higher than our study [14,42]. These results may be associated with the limited space where the calves are housed, as they do not have enough room to wear down their hooves. In our study, no cases of scissor claws or corkscrew claws were detected, which can also be considered asymmetries, probably associated with the short lifespan of the cattle sampled. Since hoof asymmetry does not usually manifest lameness in animals, observational studies based on lameness detection may underestimate the problem. Hoof deformities can be of both hereditary and non-hereditary origin, involving abnormal growth of the horn tissue of the foot and predisposing to injuries that cause pain and discomfort while walking since the weight distribution on each digit becomes unequal. At the same time, the presence of these deformations constitutes a risk factor for the development of other injuries, such as ulcers or double soles, which could explain the high prevalences obtained in this last case.

The prevalence observed of digital dermatitis in our study is lower than those reported in the literature, both for beef [43,44] and dairy cattle [32,41,42,45,46]. Given that the primary etiology is wounds, the low prevalence could be associated with the management type in the study area as well as the age of the animals (average of 10 months), where calf rearing is carried out in small facilities, resulting in a low probability of injuries.

In our study, the double sole was the second most observed lesion, with a prevalence much higher than that observed in other studies [14,33,41,45,47]. The high prevalence is challenging to justify, although it may be associated with the type of management where calf rearing occurs in small premises, where factors such as uneven floors, moisture, and environmental contamination (due to the accumulation of material—i.e., dirt—between the layers of a pinpoint defect) may lead to vascular disturbances in the corium, resulting in alterations in the dermal–epidermal junction due to the existence of uneven flooring [48]. These alterations cause a discontinuation in the production of horn tissue. Over time, these lesions heal, initiating the production of new horn tissue, resulting in a new layer forming a double sole [49]. Thus, the higher prevalence observed may be indicative of inadequate flooring for cattle in the study region as well as associated with the rapid growth of corium in calves [50].

Heel horn erosion was the most prevalent claw lesion. This pathology appears in cattle whose hooves are in contact with uneven surfaces, wet areas, and/or a high amount of manure due to poor hygiene most of the time. When hooves are continually submerged in manure, the skin and horny tissue soften, creating a conducive environment (i.e., crack) for the proliferation of bacteria, primarily *Fusobacterium necrophorum* and *Bacteroides melaninogenicus*. As the lesion progresses, pain increases, and the animal loses hoof stability, increasing the risk of developing other pathologies, such as interdigital dermatitis, due to improper weight distribution since these lesions often occur together. The fact that other authors report much lower prevalences may be associated with the studies being conducted on dairy farms where soil and bedding hygiene are more controlled [25,33,45,47]. Also, the fact that the front limbs were the most affected could be associated with the observation that the front legs dig deeper into the bedding. Therefore, the high prevalence observed, along with the high prevalence of double sole, is consistent with the predominant management practices in the study area. Furthermore, the fact that this type of lesion is more frequent in confined animals [32] than in pastured animals indicates that the quality of the flooring, as well as cleanliness and disinfection, is insufficient, aligning with the earlier-explained double sole results. In most of the literature, it is indicated that HHE appears alongside ID. Also, it has been referred to that ID lesions develop between 2 and 19 weeks, although delayed development until 2 years has been referred to [51]. The fact that in our study, ID has a low prevalence could be justified by the fact that DD lesions develop and progress very slowly, as reported elsewhere. Animals’ lifespan is shorter than the time required for the development of interdigital dermatitis as a consequence of secondary bacterial proliferation in heel erosions [52,53,54], initiation, and degeneration of tissues [55]. 

In our study, the prevalence of ulcers is lower than what has been observed by other authors [29,33,56,57]. This could be because the cattle study population sampled consists of young and growing animals since factors related to the periparturient period (calving age, primiparous/multiparous, claw lesions before or during lactation, development of mastitis) have been considered risk factors in the development of sole ulcers or sole hemorrhages [58].

The fact that 65% of sampled cattle presented some type of claw lesion indicates that the conditions in which the animals are kept need improvement. The high values of the double sole can be justified by the fact that, in the case of growing animals, cell multiplication in different tissues is higher. Furthermore, the low prevalence of ulcers can be attributed to the fact that, in the case of young animals, cell multiplication and tissue repair are higher and faster. Additionally, given the short lifespan of these animals, the time required for necrotic tissue progression to develop ulcers exceeds their lifespan. Since most studies are conducted on dairy farms during the lactation phase with older cattle than in our study, a higher prevalence of ulcers is found in dairy cattle. This suggests that these types of injuries are more common in older cattle, where such injuries require a longer period to develop. 

The absence of white-line disease in our study may be related to the mean age of the study population, as previously explained for ulcers [48].

As previously explained, digital dermatitis is related to heel horn erosion [52,53,54]. The fact is that the prevalence of digital dermatitis is low. It may be related to the development period of the lesion, which is longer than the lifespan of the calves. In addition, the low prevalence observed could be explained by the fact that cattle sampled are raised in small premises with limited movement, and the bedding does not contain stones or other objects that could cause injuries to the epidermis.

In our study, sex and age do not appear to have a clear influence on the presence of claw lesions (*p* > 0.05), although a higher percentage is observed in females between 7 and 12 months. Males seem to be more likely to develop claw problems, probably associated with higher weight compared to females with similar characteristics [3]. However, this result must be carefully interpreted since the number of female cattle sampled was double that of male cattle. 

Although in our study, animals aged 13–18 months and those over 18 months have a low prevalence, age seems to influence the development of claw lesions [59]. Increased age implies greater growth of the animal and, therefore, its weight, influencing the development of claw lesions due to increased pressure on the sole [31], as described above. This statement aligns with the claw lesions found in this study because, for the same carcass weight category, the prevalence of claw lesions is higher as age increases. Regarding carcass weight, the presence of claw lesions does not appear to influence the growth of the cattle studied, contrary to what was reported elsewhere [60]. Considering the inherent differences in the growth of males and females, it would be expected that cattle with higher weight within each age group have a greater predisposition to claw lesions, as explained earlier. Given that claw lesions do not influence carcass weight, it is justifiable to conclude that they also have no impact on both carcass conformation and fat deposition. When a bovine exhibits lameness, it not only hinders mobility but also causes significant pain. In such circumstances, food intake decreases, affecting weight (as well as milk production in the case of dairy farms). Although the prevalence of lesions observed is high, the lack of influence on key production characteristics (weight, classification, and fat deposition in the carcass) indicates that these lesions, while present, do not have a significant clinical expression. However, even if the animal does not show signs of discomfort, it does not feel it. Therefore, the care of all aspects related to claw welfare (i.e., bedding, laying area, among others) should always be optimized. 

Given that our work is an observational study at the slaughterhouse, further research is necessary to evaluate the existence of clinical signs at farms and their potential impact on production parameters.

The results highlight the need to improve housing and bedding conditions to guarantee proper cattle welfare. The authors remark that the absence of claw lesions on the production parameters must be interpreted carefully since the study sample mainly consisted of young cattle slaughtered, paying attention to the particularities of cattle management in the study region. Furthermore, another limitation of this study is that it has not investigated other factors related to the development of claw lesions, such as farm conditions, management, or feeding. Thus, the high prevalence of hoof lesions could have both a clinical and production impact on those farms where beef cattle are slaughtered at an older age.

## 5. Conclusions

The animal prevalence of claw lesions in the present study is high (65.8%), with the main lesions being of a non-infectious nature (heel horn erosion, double sole, and asymmetric claws). The results obtained are consistent with the type of cattle production in the study region, as previously described, with special importance given to the bedding characteristics. 

The influence of sex and age did not affect the presence of claw lesions, although there seems to be a greater predisposition in females and a higher prevalence as age increases. In the latter case, it is probably associated with increased weight. 

The influence of claw lesions on production characteristics (weight, classification, and fat deposition of carcasses) was also not evident.

## Figures and Tables

**Table 1:** Distribution of claw lesions.

	N	%
Prevalence ^1^ of claw lesions	226	65.8
*Number of hooves affected* ^2^		
One hoof	44	19.4
Two hooves	168	74.3
Three hooves	5	2.2
Four hooves	9	3.9
*Number of claw lesions per animal* ^2^		
One lesion	180	79.6
Two lesions	39	17.2
Three lesions	7	3.1
Four lesions	0	0
Total cattle with claw lesions in the front limbs ^2^	214	94.6
Total cattle with claw lesions in the hind limbs ^2^	33	14.6
Total cattle with claw lesions in front and hind limbs ^2^	21	9.3
*Presence of claw lesions by limb location* ^2^		
Right front limb	181	80.1
Left front limb	204	90.2
Right hind limb	21	9.3
Left hind limb	25	11.1

^1^: Refers to animal prevalence. ^2^: Based on 226 cattle with at least one claw lesion.

**Table 2:** Prevalence ^1^ and location of claw lesions.

Claw Lesion	N	%	Front Limbs		Hind Limbs		P^2^
			Left	Right	Left	Right	
Asymmetric claws	37	16.37	29	14	6	2	*p* < 0.001
Concave dorsal wall	6	2.65	2	3	1	3	ns ^a^
Digital dermatitis	3	1.32	2	1	0	0	ns
Interdigital dermatitis	1	0.44	1	0	1	0	ns
Double sole	103	45.57	101	99	9	9	*p* < 0.001
Heel horn erosion	112	49.55	104	105	12	16	*p* < 0.001
Axial horn erosion	1	0.44	1	0	0	0	ns
Interdigital phlegmon	2	0.88	1	2	1	1	ns
Sole ulcer	5	2.21	5	1	3	0	ns ^a^
Toe ulcer	6	2.65	4	5	0	1	ns
Toe necrosis	5	2.21	2	3	2	2	ns ^a^

ns: not significant. ^1^: results are expressed by affected cattle (n = 226). P^2^ indicates statistical differences only between forelimbs and hindlimbs. ^a^: the *p*-value for concave dorsal wall, sole ulcer, and toe necrosis was *p* < 0.001. Since lesions were rarely recorded, the significance is purely mathematical, not biological. Consequently, the *p*-value for the three lesions was presented as not significant (ns).

**Table 3:** Distribution (n) of hot carcass weight by sex and age according to the absence or presence of claw lesions.

Age (CAT)	Hot Carcass Weight	Female		Male	
		Absence	Presence	Absence	Presence
≤6 months	≤100 Kg	1	0	0	0
	>100–150≤ Kg	0	1	0	1
	>150–200≤ Kg	3	3	0	0
	>200–250≤ Kg	0	0	1	1
	>250–300≤ Kg	0	0	1	0
	>300–350≤ Kg	0	0	0	0
	>350–400≤ Kg	0	0	0	0
≤7–12≥ months	≤100 Kg	0	0	1	1
	>100–150≤ Kg	1	2	1	2
	>150–200≤ Kg	32	55	5	15
	>200–250≤ Kg	26	34	16	29
	>250–300≤ Kg	0	3	10	25
	>300–350≤ Kg	0	0	1	6
	>350–400≤ Kg	0	0	0	2
≤13–18≥ months	≤100 Kg	0	0	0	0
	>100–150≤ Kg	0	0	0	0
	>150–200≤ Kg	3	5	1	3
	>200–250≤ Kg	10	15	0	4
	>250–300≤ Kg	0	6	1	2
	>300–350≤ Kg	0	1	1	3
	>350–400≤ Kg	0	0	0	1
≥19 months	≤100 Kg	0	0	0	0
	>100–150≤ Kg	0	0	0	0
	>150–200≤ Kg	0	0	0	0
	>200–250≤ Kg	0	2	0	0
	>250–300≤ Kg	0	1	0	0
	>300–350≤ Kg	1	2	1	0
	>350–400≤ Kg	0	0	0	1

## Data Availability

Data are contained within the article and Supplementary Materials.

## Supplementary Materials

The following supporting information can be downloaded at: https://www.mdpi.com/article/10.3390/ani14030514/s1, The supporting information provides details on cattle claw lesions by sex, age, hot carcass weight, carcass classification, and fat coverage.
