# Supplementary material for: Evaluation of Claw Lesions in Beef Cattle Slaughtered in Northern Portugal: A Preliminary Study
Source: Animals (Basel). 2024 Feb 4;14(3):514. doi: 10.3390/ani14030514 (PMC10854553; doi:10.3390/ani14030514)
Supplement: Supplementary file 1 [file animals-14-00514-s001.zip › animals-2825183-supplementary.pdf]

Suplementary

Prevalence (%) of cattle affected with asymmetric claws by sex (a), age (b), hot carcass weight (c), carcass classification (d) and fat coverage (e)

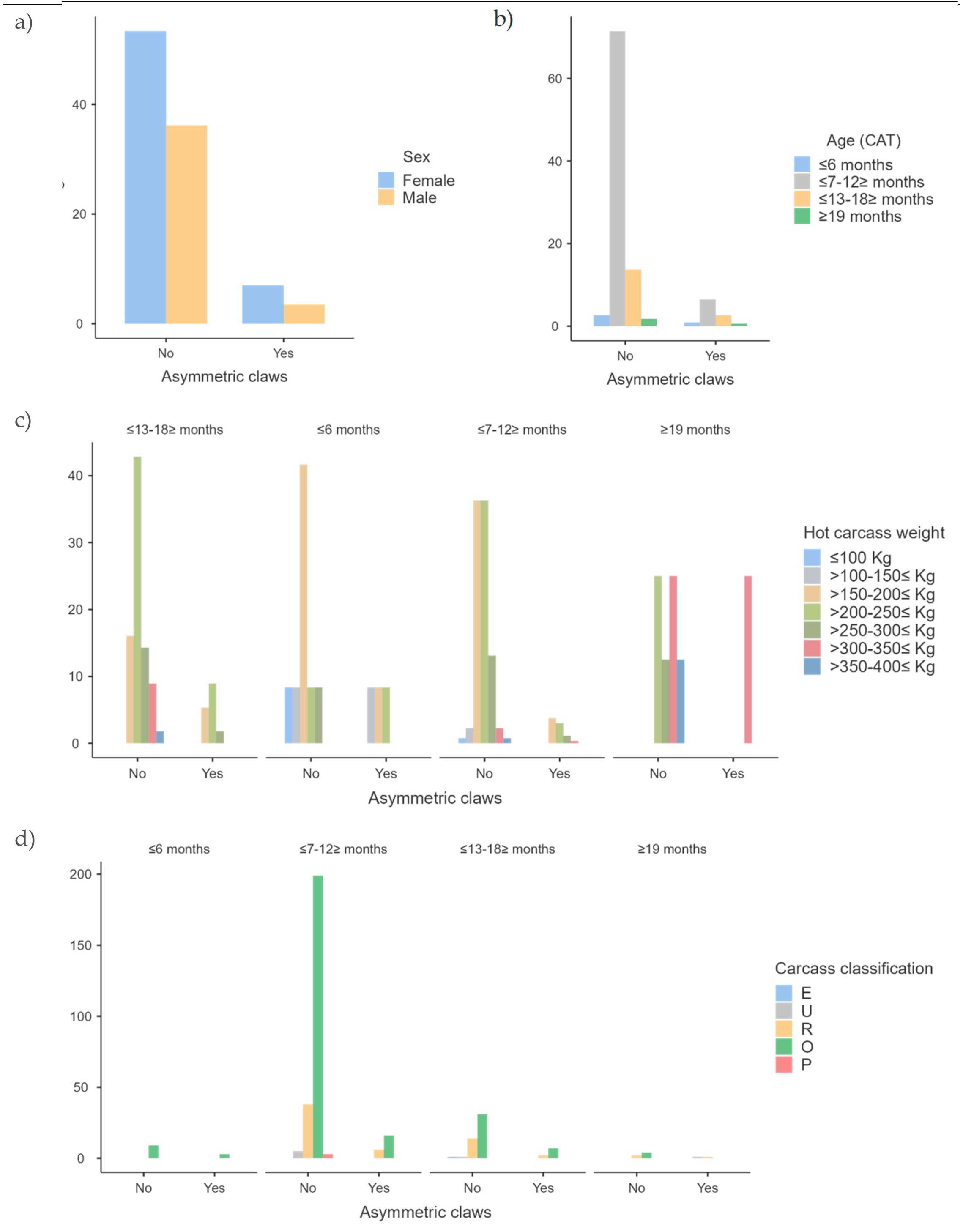

e)

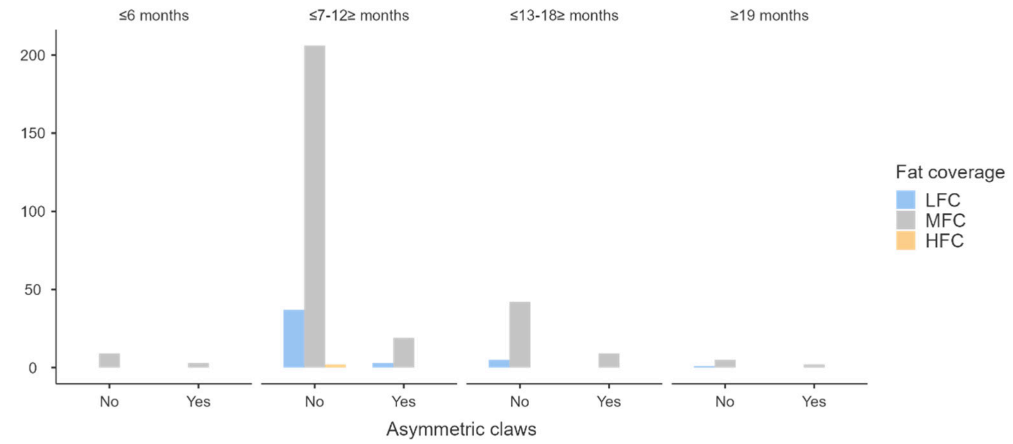

Prevalence (%) of cattle affected by concave dorsal wall by sex (a), age (b), hot carcass weight (c), carcass classification (d) and fat coverage (e)

a)

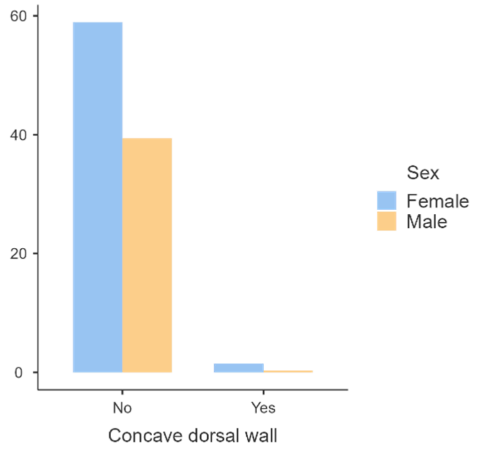

b)

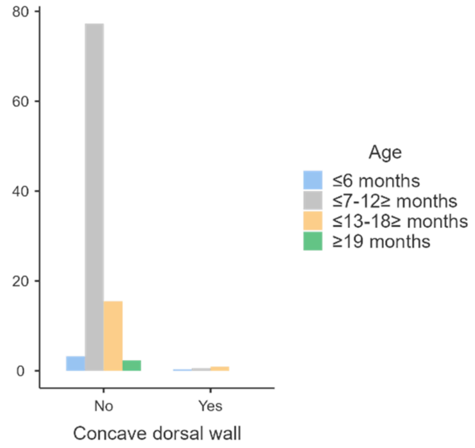

c)

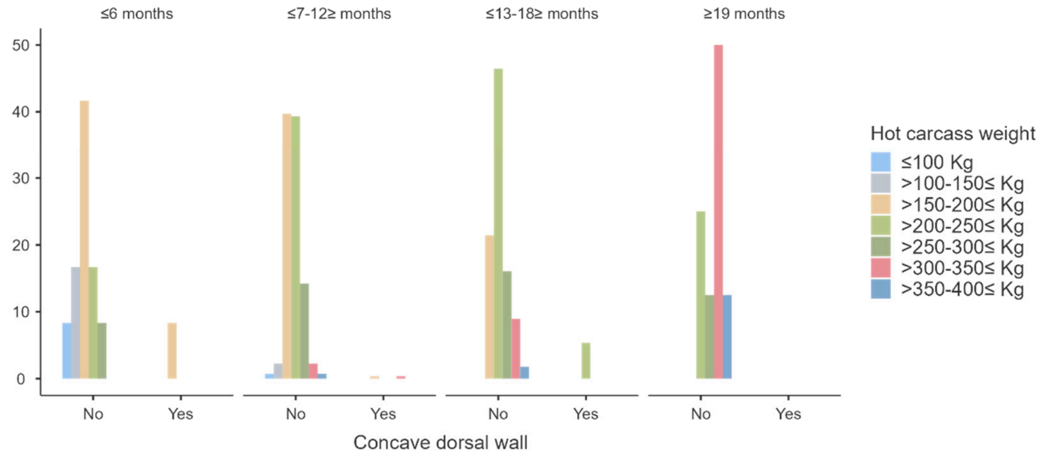

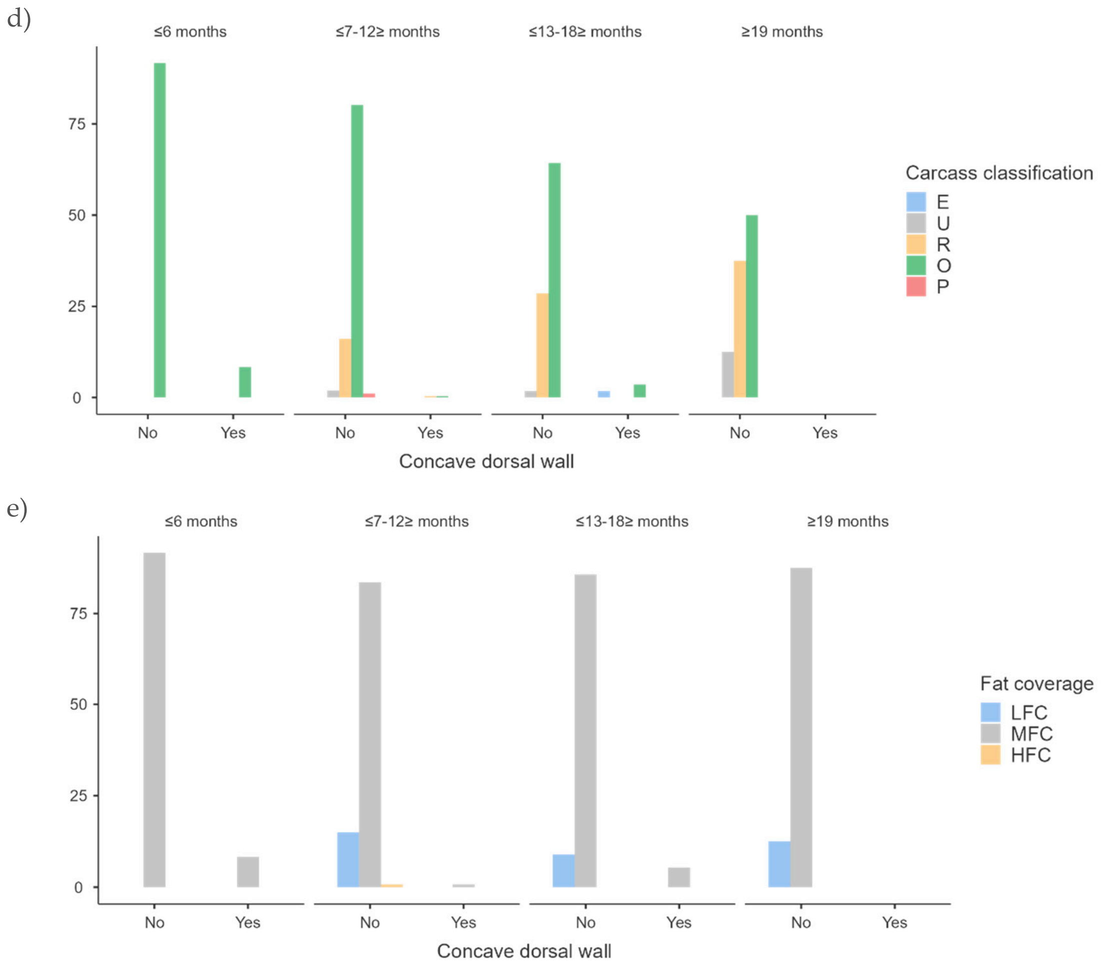

Prevalence (%) of cattle affected by digital dermatitis by sex (a), age (b), hot carcass weight (c), carcass classification (d) and fat coverage (e)

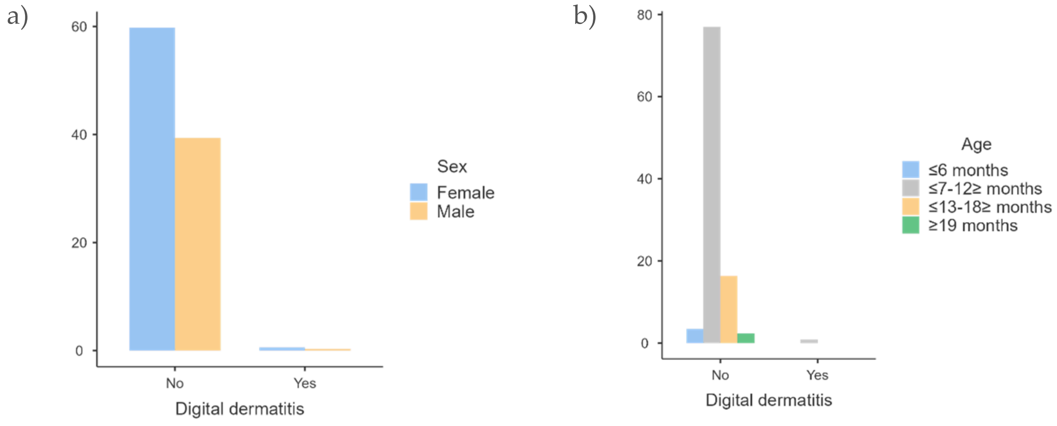

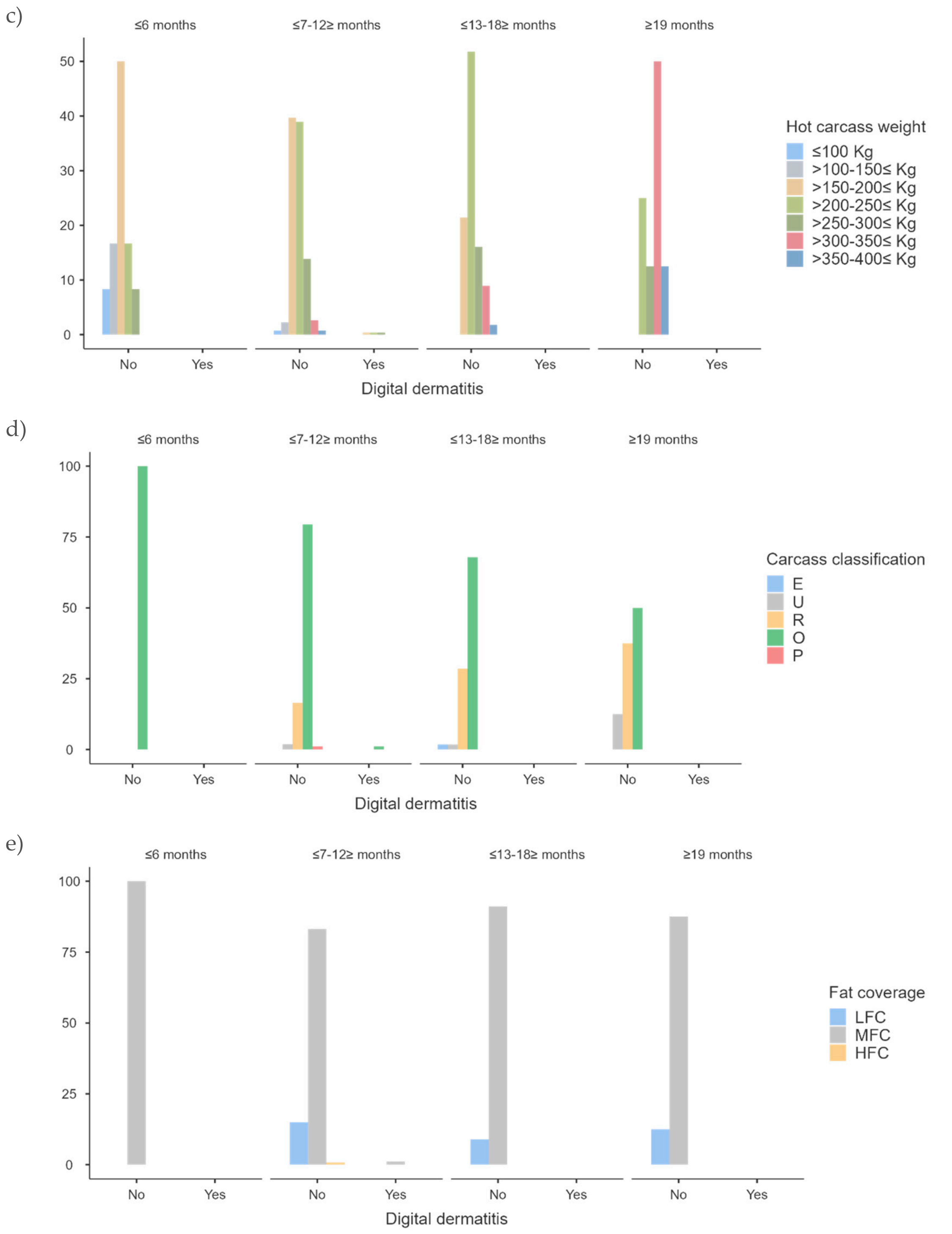
c)
≤6 months
≤7-12≥ months
≤13-18≥ months
≥19 months
0
10
20
30
40
50
No
Yes
Digital dermatitis
Hot carcass weight
≤100 Kg
>100-150≤ Kg
>150-200≤ Kg
>200-250≤ Kg
>250-300≤ Kg
>300-350≤ Kg
>350-400≤ Kg
d)
≤6 months
≤7-12≥ months
≤13-18≥ months
≥19 months
0
25
50
75
100
No
Yes
Digital dermatitis
Carcass classification
E
U
R
O
P
e)
≤6 months
≤7-12≥ months
≤13-18≥ months
≥19 months
0
25
50
75
100
No
Yes
Digital dermatitis
Fat coverage
LFC
MFC
HFC

Prevalence (%) of cattle affected by interdigital / superficial dermatitis by sex (a), age (b), hot carcass weight (c), carcass classification (d) and fat coverage (e)

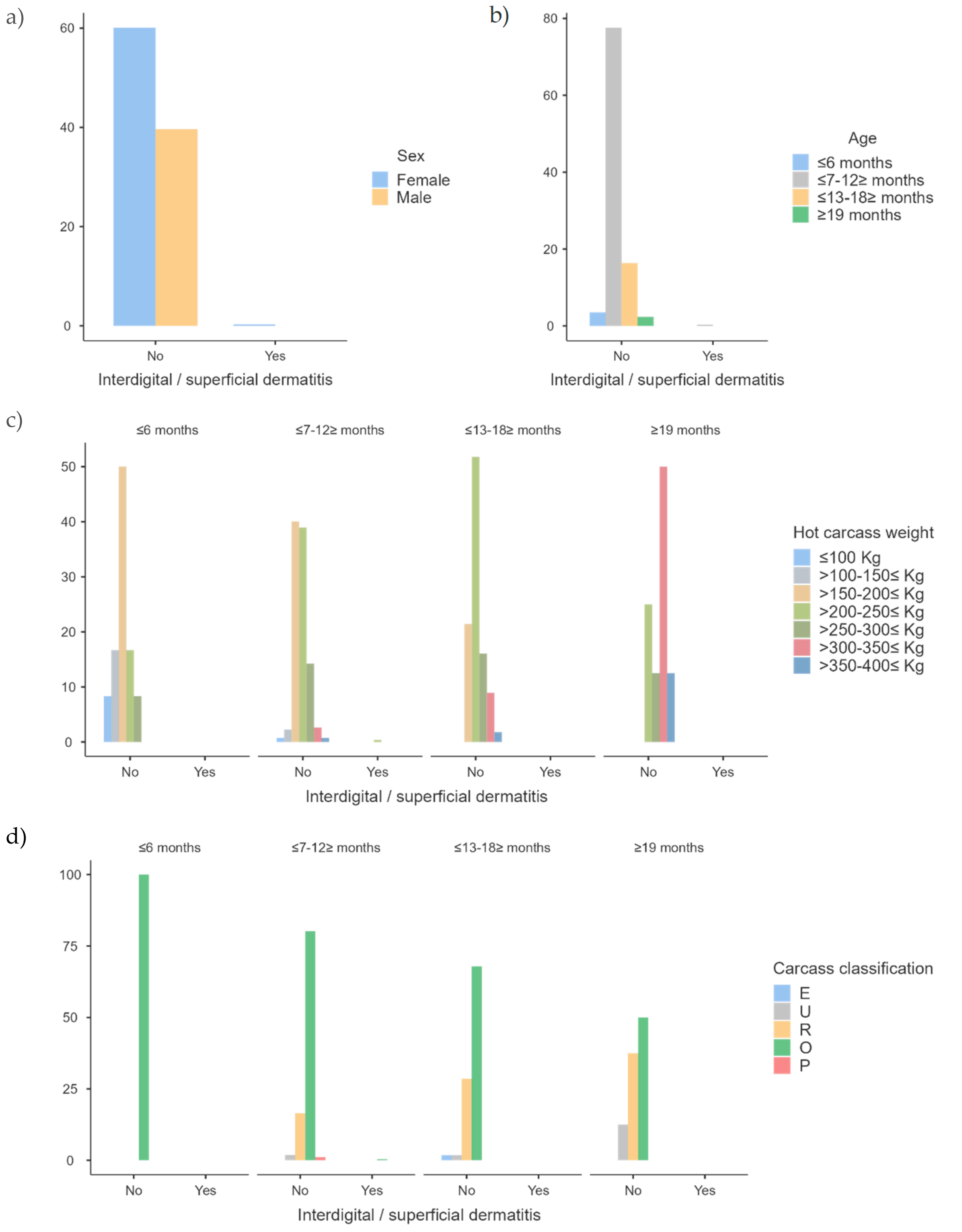

e)

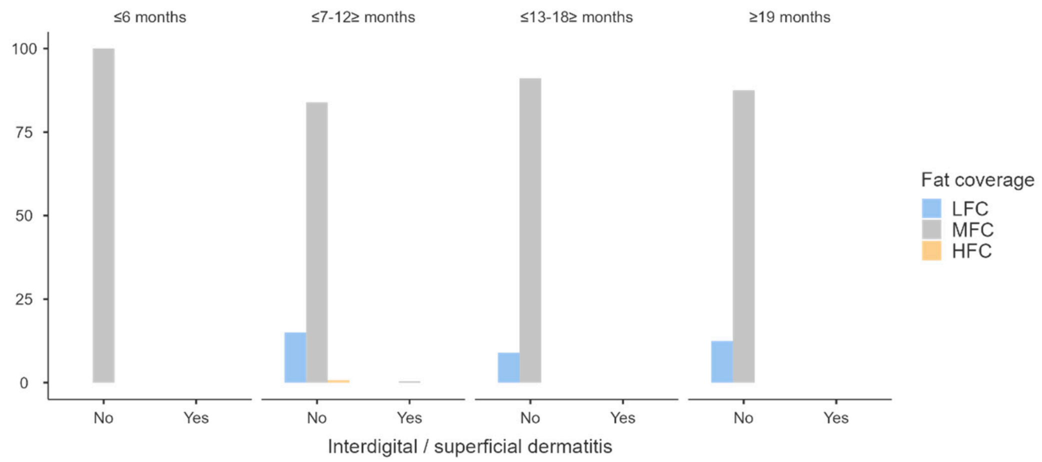

Prevalence (%) of cattle affected by double sole by sex (a), age (b), hot carcass weight (c), carcass classification (d) and fat coverage (e)

a)

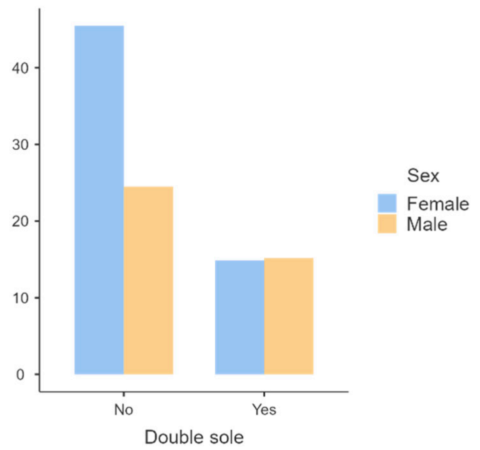

b)

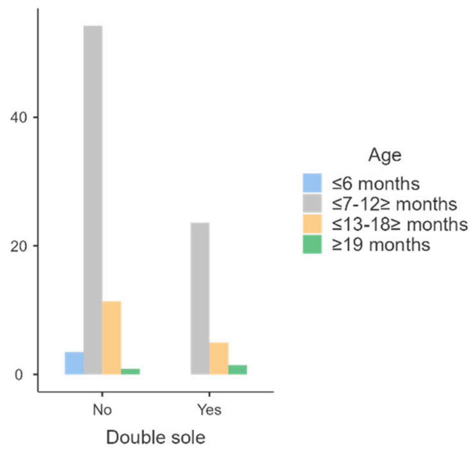

c)

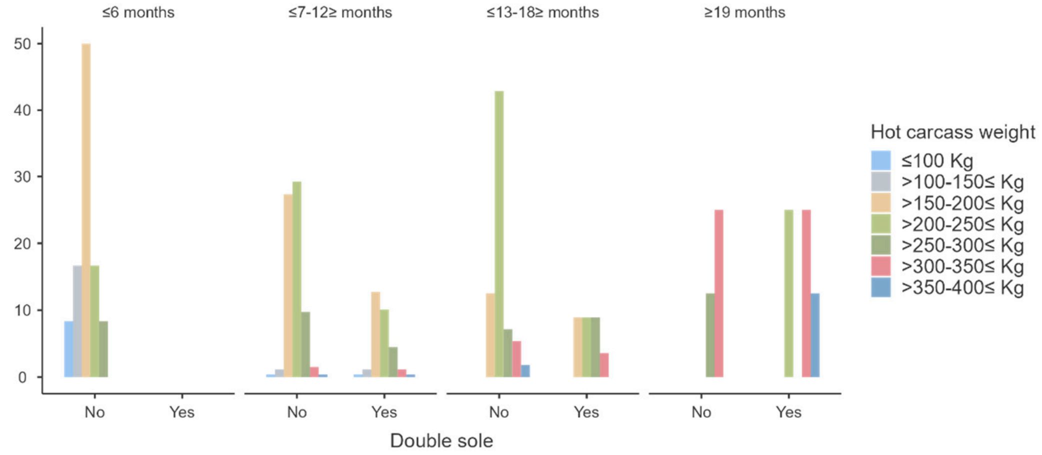

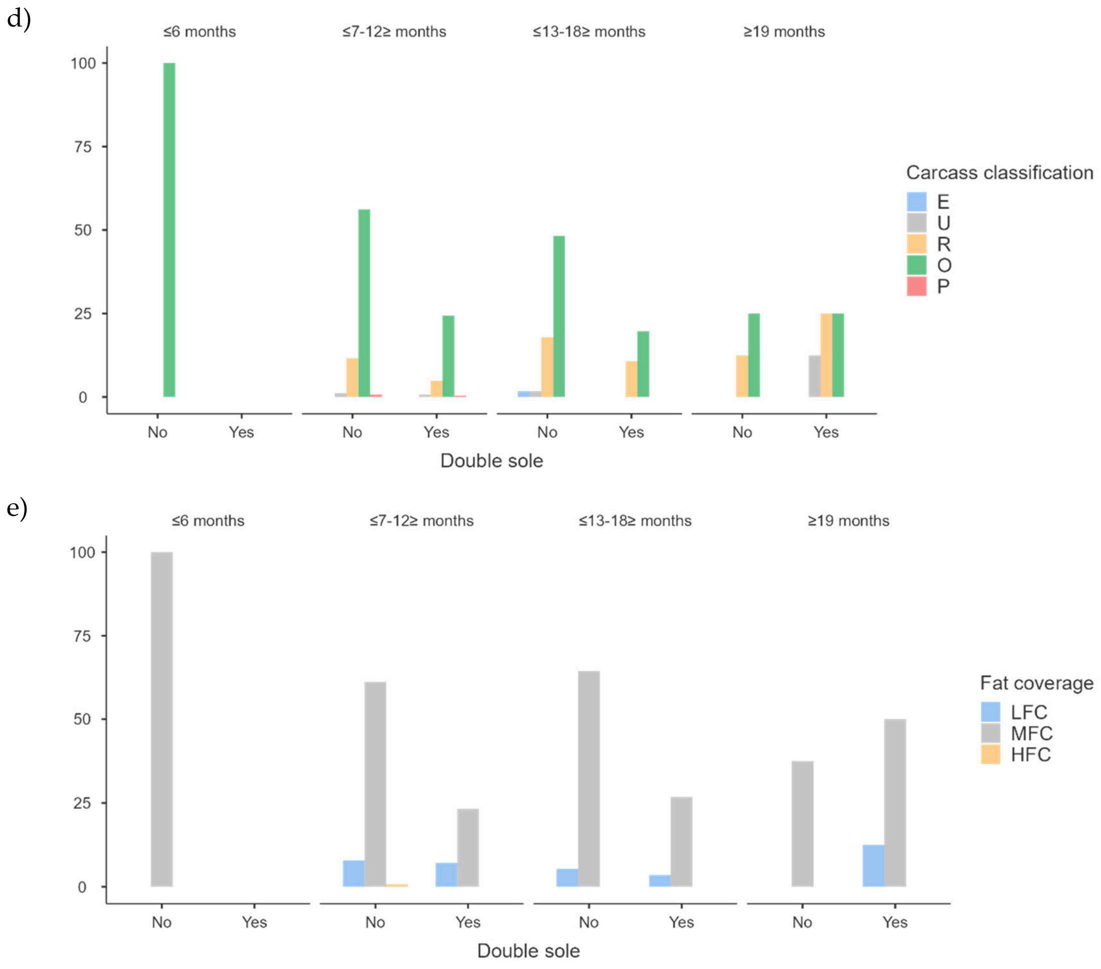

Prevalence (%) of cattle affected by heel horn erosion by sex (a), age (b), hot carcass weight (c), carcass classification (d) and fat coverage (e)

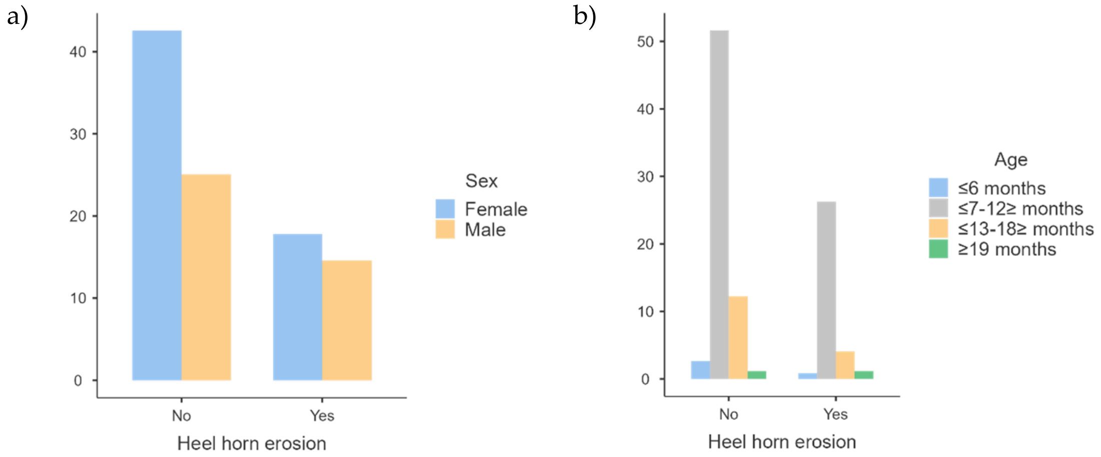

c)

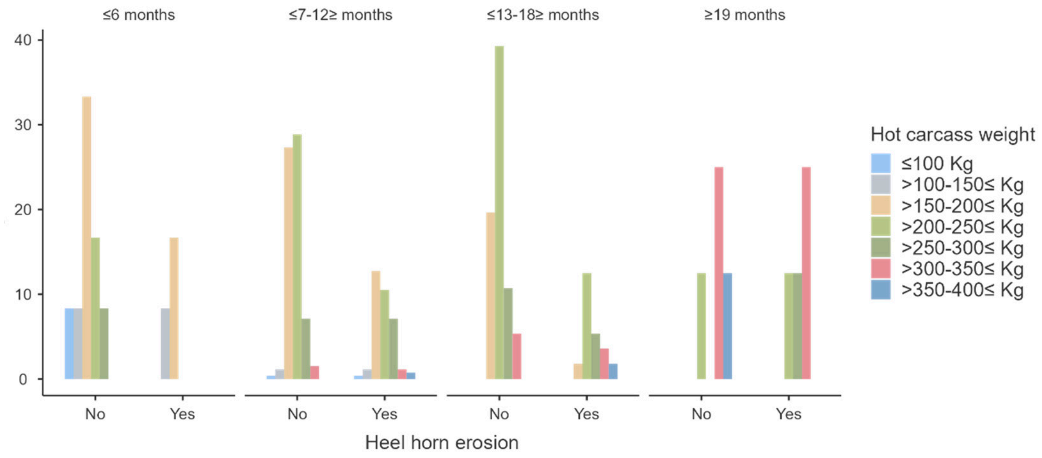
≤6 months
≤7-12≥ months
≤13-18≥ months
≥19 months
Hot carcass weight
≤100 Kg
>100-150≤ Kg
>150-200≤ Kg
>200-250≤ Kg
>250-300≤ Kg
>300-350≤ Kg
>350-400≤ Kg
Heel horn erosion

d)

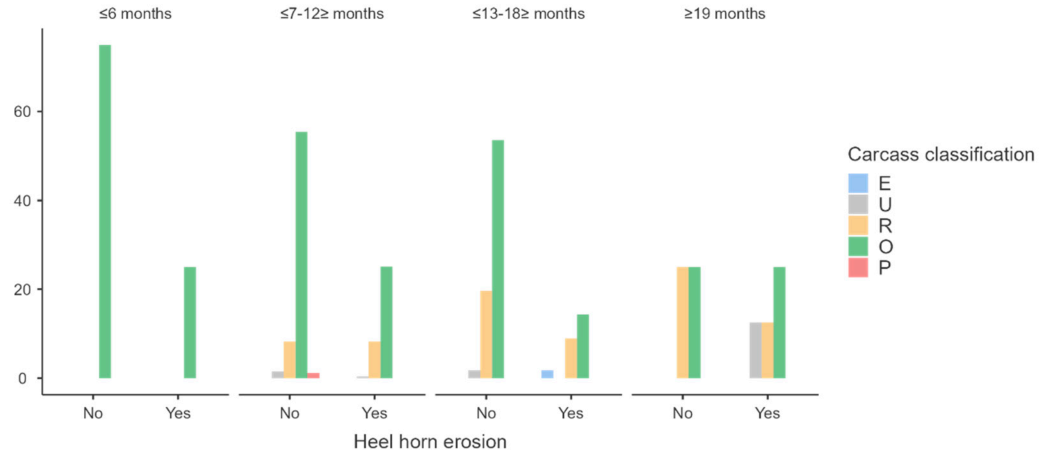
≤6 months
≤7-12≥ months
≤13-18≥ months
≥19 months
Carcass classification
E
U
R
O
P
Heel horn erosion

e)

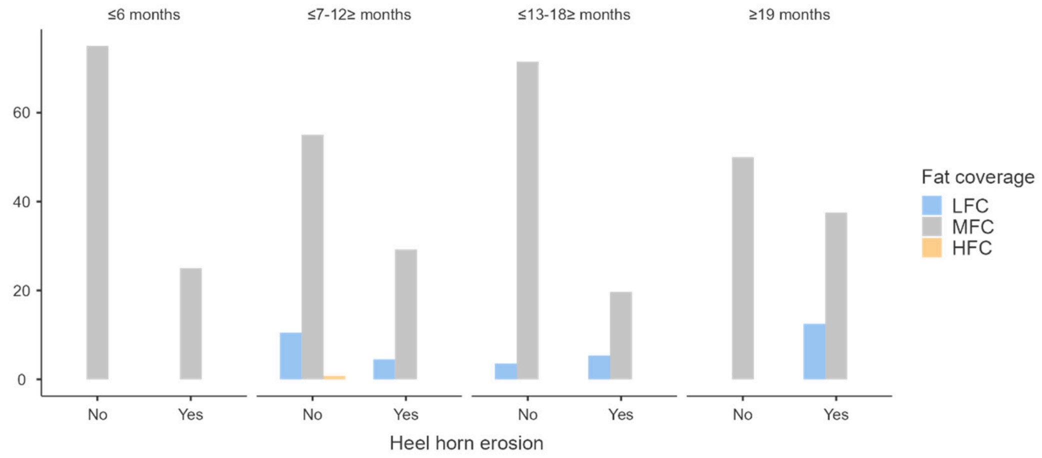
≤6 months
≤7-12≥ months
≤13-18≥ months
≥19 months
Fat coverage
LFC
MFC
HFC
Heel horn erosion

Prevalence (%) of cattle affected by sole ulcer by sex (a), age (b), hot carcass weight (c), carcass classification (d) and fat coverage (e)

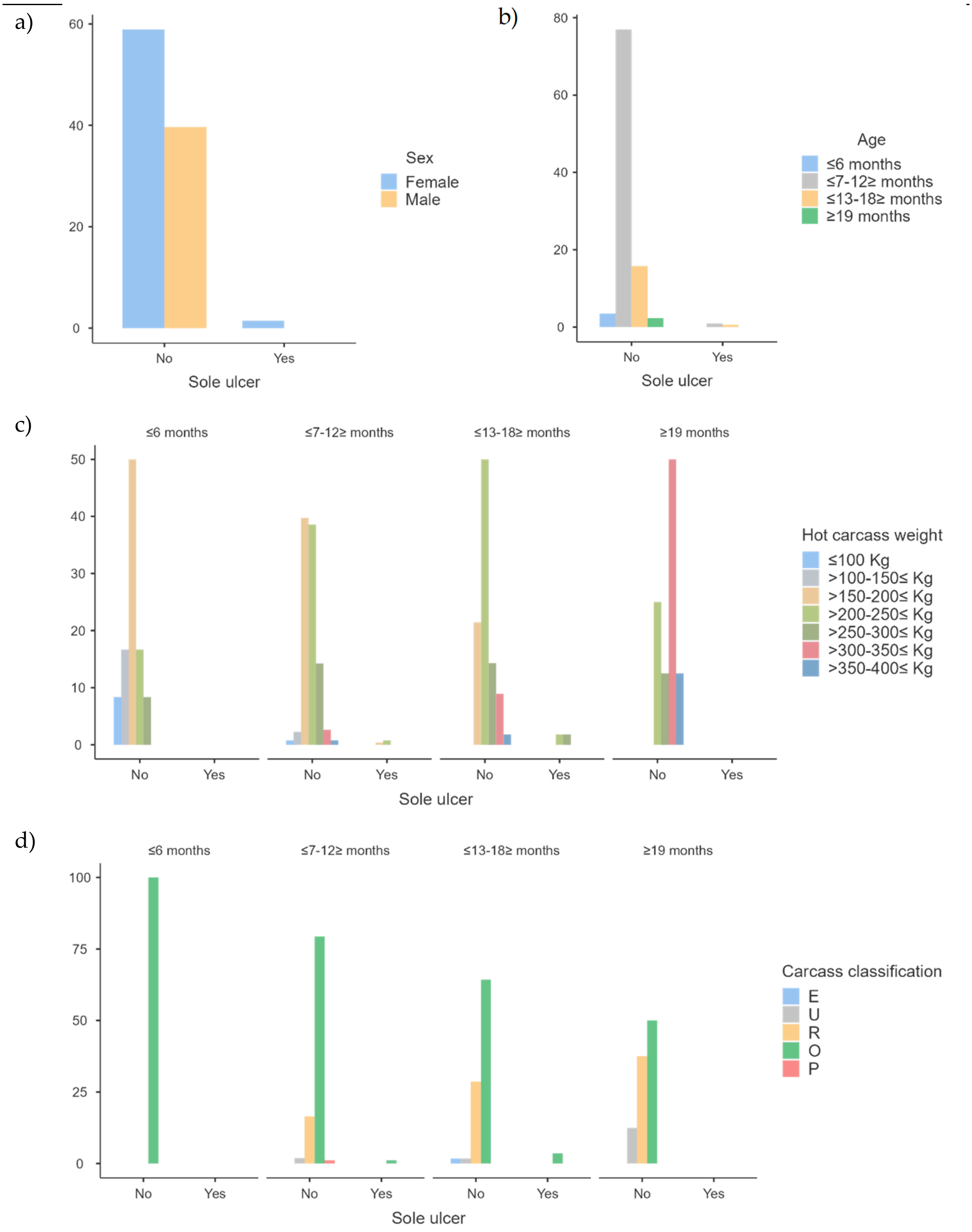

e)

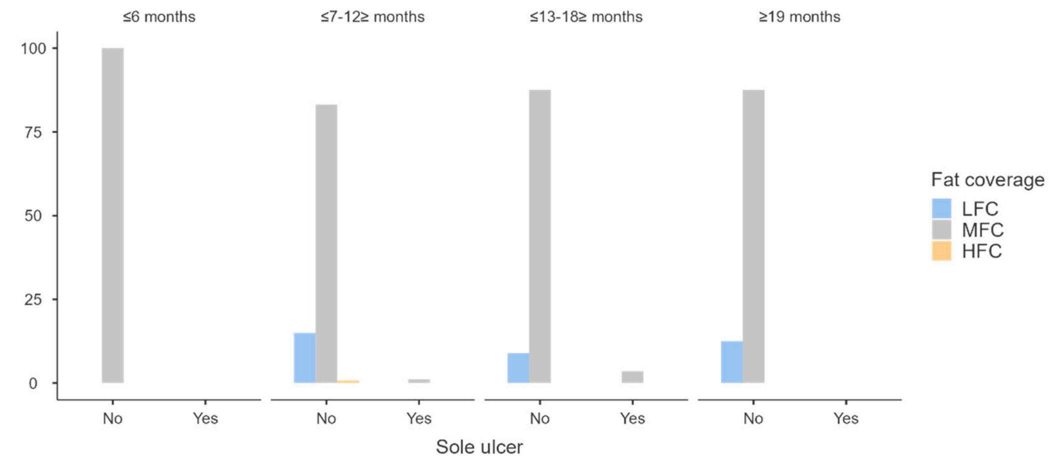

Prevalence (%) of cattle affected by toe ulcer by sex (a), age (b), hot carcass weight (c), carcass classification (d) and fat coverage (e)

a)

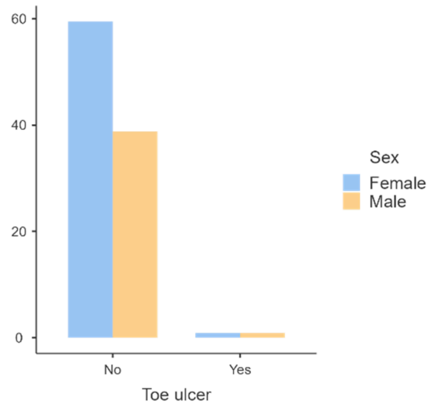

b)

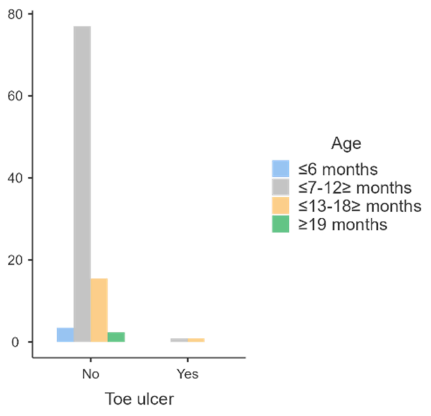

c)

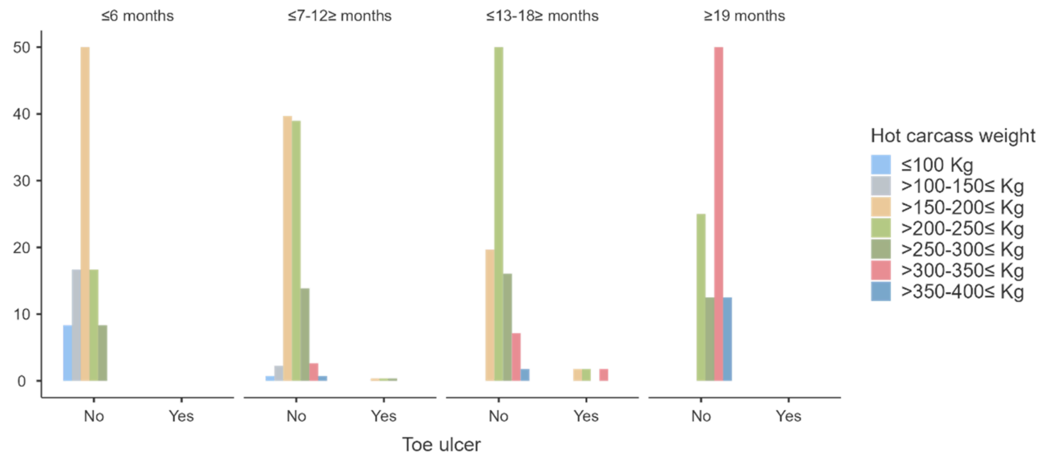

d)

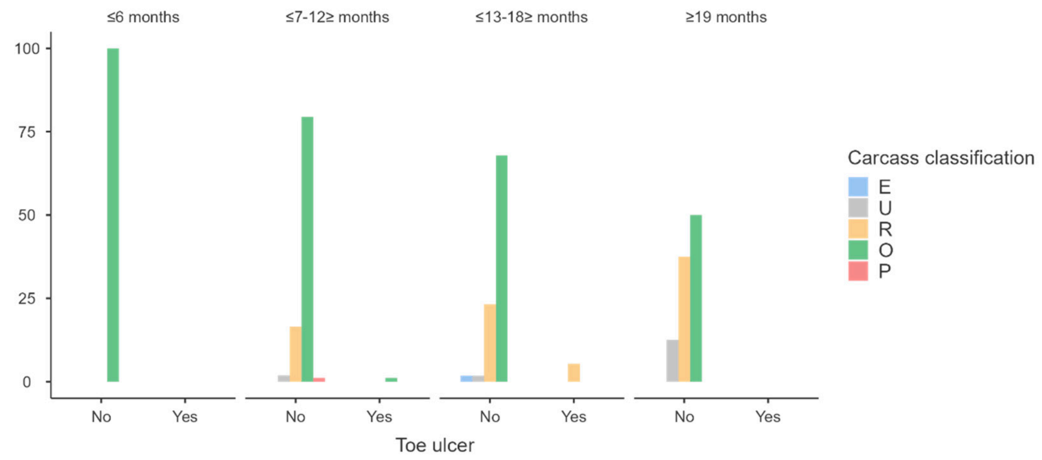

e)

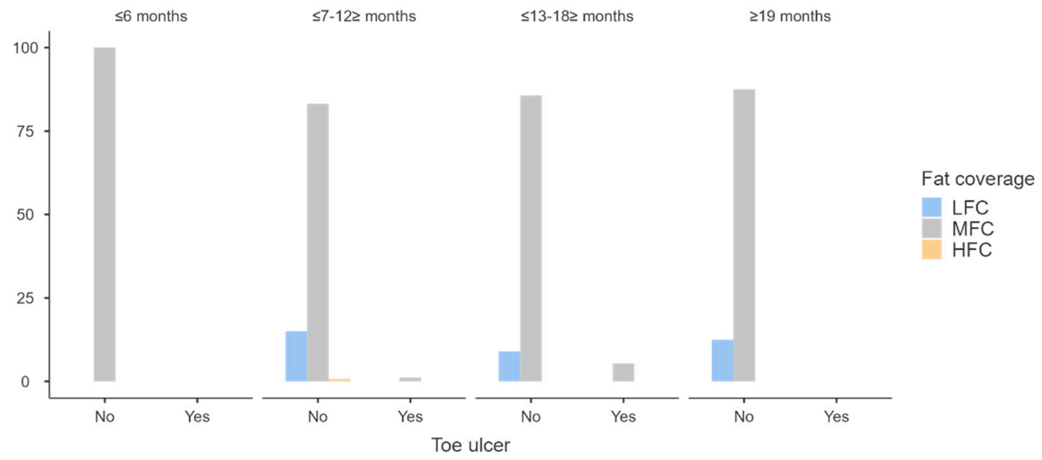

Prevalence (%) of cattle affected by toe necrosis by sex (a), age (b), hot carcass weight (c), carcass classification (d) and fat coverage (e)

a)

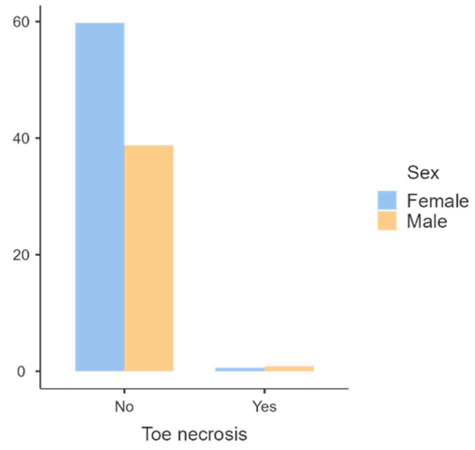

b)

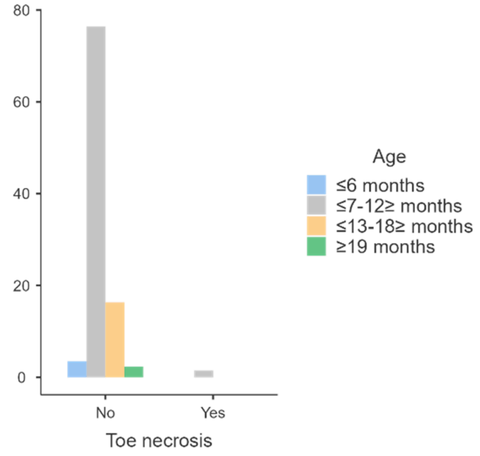

c)

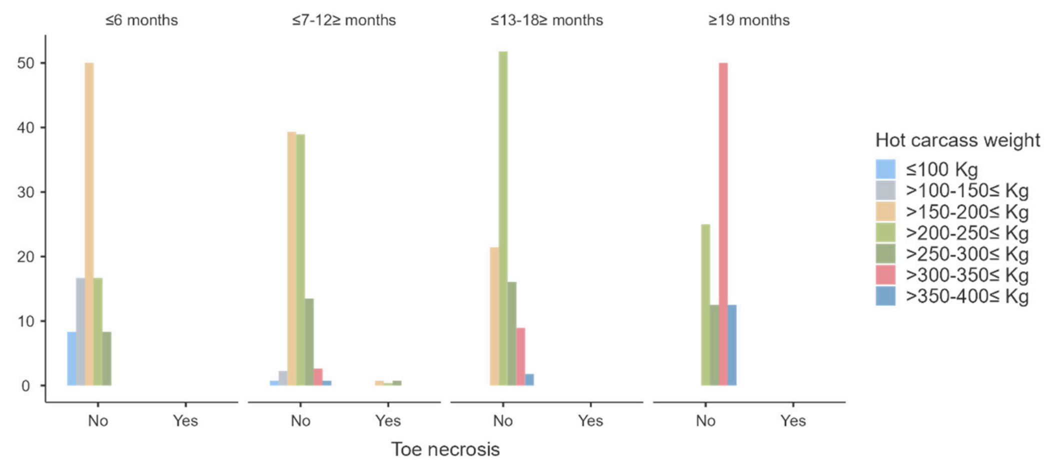
≤6 months
≤7-12≥ months
≤13-18≥ months
≥19 months
0
10
20
30
40
50
No
Yes
Toe necrosis
Hot carcass weight
≤100 Kg
>100-150≤ Kg
>150-200≤ Kg
>200-250≤ Kg
>250-300≤ Kg
>300-350≤ Kg
>350-400≤ Kg

d)

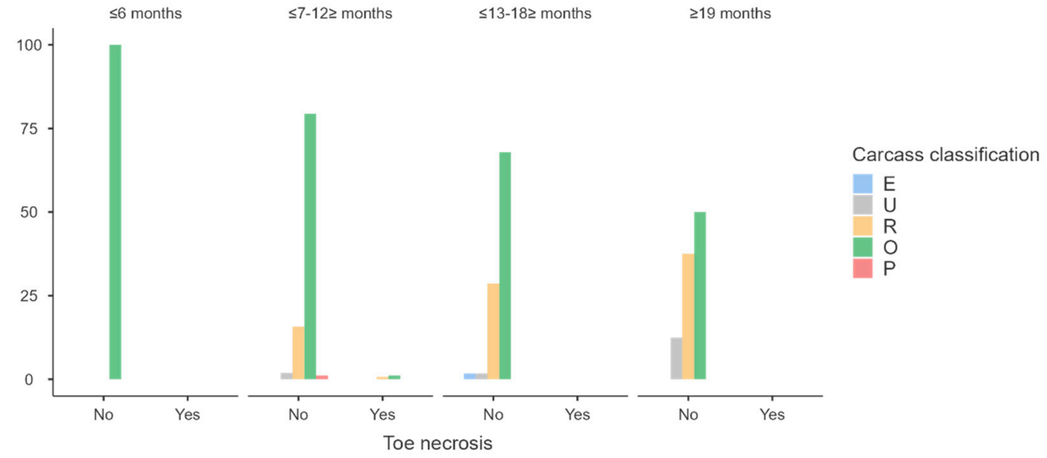
≤6 months
≤7-12≥ months
≤13-18≥ months
≥19 months
0
25
50
75
100
No
Yes
Toe necrosis
Carcass classification
E
U
R
O
P

e)

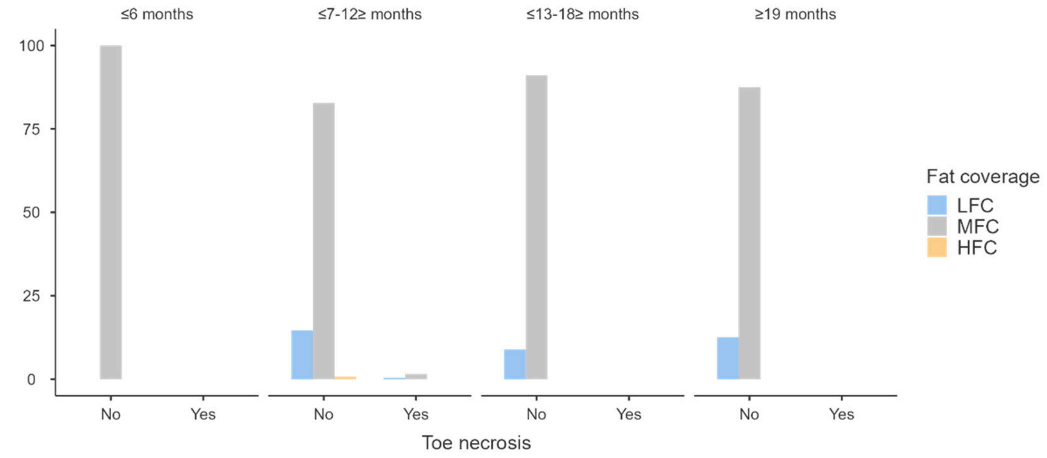
≤6 months
≤7-12≥ months
≤13-18≥ months
≥19 months
0
25
50
75
100
No
Yes
Toe necrosis
Fat coverage
LFC
MFC
HFC
